# Chronic kidney disease in patients at high risk of cardiovascular disease in the United Arab Emirates: A population-based study

**DOI:** 10.1371/journal.pone.0199920

**Published:** 2018-06-27

**Authors:** S Al-Shamsi, D Regmi, R D Govender

**Affiliations:** 1 Department of Internal Medicine, College of Medicine and Health Sciences, United Arab Emirates University, Al Ain, United Arab Emirates; 2 Department of Family Medicine, College of Medicine and Health Sciences, United Arab Emirates University, Al Ain, United Arab Emirates; Nagoya University, JAPAN

## Abstract

Chronic kidney disease has become an increasingly significant clinical and public health issue, accounting for 1.1 million deaths worldwide. Information on the epidemiology of chronic kidney disease and associated risk factors is limited in the United Arab Emirates. Therefore, this study aimed to evaluate the incidence and causes of chronic kidney disease stages 3–5 in adult United Arab Emirates nationals with or at high risk of cardiovascular disease. This retrospective study included 491 adults with or at high risk of cardiovascular disease (diabetes mellitus or associated clinical disease) who attended outpatient clinics at a tertiary care hospital in Al-Ain, United Arab Emirates. Estimated glomerular filtration rate was assessed every 3 months from baseline to June 30, 2017. Chronic kidney disease stages 3–5 were defined as an estimated glomerular filtration rate < 60 mL/min/1.73 m2 for ≥ 3 months. Multivariable Cox's proportional hazards analysis was used to determine the independent risk factors associated with developing chronic kidney disease stages 3–5. The cumulative incidence of chronic kidney disease stages 3–5 over a 9-year period was 11.4% (95% confidence interval 8.6, 14.0). The incidence rate of these disease stages was 164.8 (95% confidence interval 121.6, 207.9) per 10,000 person-years. The independent risk factors for developing chronic kidney disease stages 3–5 were older age, history of coronary heart disease, history of diabetes mellitus, and history of smoking. These data may be useful to develop effective strategies to prevent chronic kidney disease development in high-risk United Arab Emirates nationals.

## Introduction

During recent decades, the United Arab Emirates (UAE) has undergone rapid economic growth and modern infrastructure development, following the discovery of oil. The shift from a semi-nomadic lifestyle to an urbanized civilization has resulted in a substantial rise of chronic diseases [1].

The prevalence of chronic kidney disease (CKD) has increased dramatically over the past two decades, with 13.4% of the population affected worldwide; the majority of the cases are CKD stages 3–5 [2]. In a recent study, the prevalence of these disease stages among UAE nationals is 4.6% in males and 2.8% in females [3]. These results may be underestimated [4], particularly if correlated with the high prevalence of known CKD risk factors in the UAE. Hypertension (HTN) plays a central role in the development of CKD [5,6] and its prevalence has increased in the UAE over the last 20 years [7]. The prevalence of type 2 diabetes mellitus (DM), another major risk factor for CKD [8,9], among UAE nationals is 29% [10]. Similarly, the presence of other cardiovascular risk factors such as obesity, dyslipidemia, and smoking in the UAE population is higher as compared to developed countries [11,12]. It is widely accepted that the rate of CKD progression is influenced by these preventable cardiovascular risk factors [13–17] and is accelerated when multiple risk factors are present in an individual [18–20].

While the incidence of CKD in the UAE and other Arab countries is not known [21], establishing local data would help better understand the epidemiological characteristics of CKD in the high-risk population of this region. Therefore, the objective of this retrospective cohort study was to determine the incidence of CKD stages 3–5 and to identify risk factors associated with developing CKD in adult UAE nationals with or at high risk of cardiovascular disease (CVD).

## Materials and methods

### Study setting

This study involved a retrospective, ambulatory electronic medical record (EMR) review of patients who presented to Tawam Hospital outpatient clinics in Al Ain, between January 1, 2008, and December 31, 2008. Al-Ain is a city in the UAE, with an estimated population of 650,000, of which 30% are UAE nationals [22]. Healthcare for UAE nationals is delivered through a publicly funded health care system. In Al-Ain, tertiary care services are provided in two public hospitals, namely, Tawam and Al-Ain Hospitals. Tawam Hospital is a 461-bed facility managed by Abu Dhabi Health Services Company (SEHA) and Johns Hopkins International. It has approximately 300,000 patient visits annually, the majority of whom are UAE nationals [23].

Cerner EMR management system was implemented at Tawam Hospital in January 2008, following which all patient medical records are available electronically. Ethical approval for this study was obtained from Tawam Hospital, and the United Arab Emirates University research and ethics board (IRR536/17). Informed consent was waived because patient records and information were anonymized and de-identified prior to analysis.

### Subjects and procedures

The study population comprised of 544 consecutive patients who had either CVD or a high CVD risk, seen at primary care and specialty outpatient clinics at Tawam Hospital. The specialty clinics included obstetrics and gynecology, internal medicine and surgery, and their respective subspecialty clinics. Inclusion criteria for this study were UAE nationals ≥ 20 years of age and diagnosed with either of the following conditions: HTN or pre-hypertension, coronary heart disease (CHD), vascular disease, DM or prediabetes, smoking, dyslipidemia, being overweight or obese.

**Fig 1:**
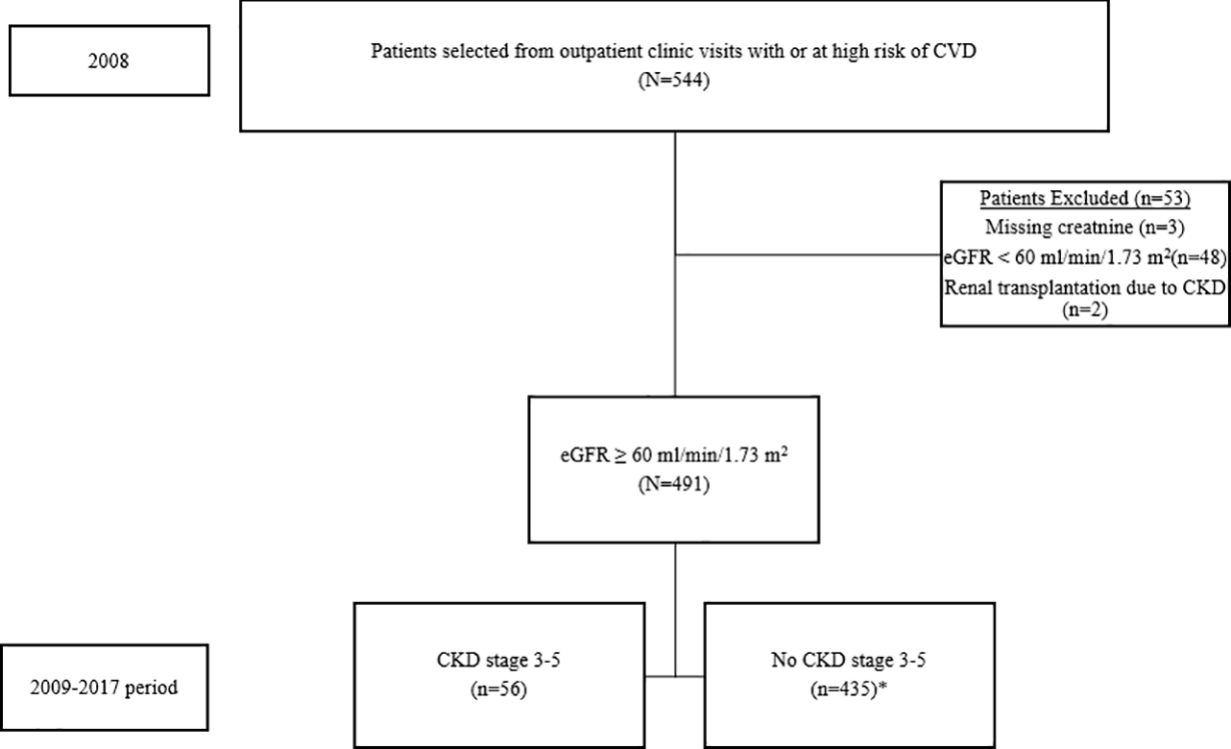
Flow diagram of subjects. CVD; cardiovascular disease, CKD; chronic kidney disease, eGFR; estimated glomerular filtration rate. *Included 6 subjects lost to follow up.

Of the 544 patients who met the inclusion criteria, 53 patients were excluded (48 patients had an estimated glomerular filtration rate (eGFR) of < 60 mL/min/1.73 m^2^, 2 patients were renal transplant recipients and 3 had serum creatinine levels missing) (Fig 1). A total of 491 subjects with eGFR ≥ 60 mL/min/1.73 m^2^ were finally included. The eGFR was repeatedly assessed for each subject every 3 months from baseline to June 30, 2017. Those with missing repeat serum creatinine during the follow-up period were excluded from the Cox's proportional hazards analysis (representing 1.10% of the initial cohort). Among the 491 eligible subjects, the data for serum triglyceride (TG) and serum glycosylated Hemoglobin, Type A1C (HbA1c) at baseline were missing in 1.2% and 3.1% of subjects, respectively.

### Definitions

Body mass index (BMI) was calculated as weight (kg) divided by height (m^2^). Overweight and obesity were defined as BMI 25–29.9 kg/m^2^ and ≥ 30 kg/m^2^ respectively. Prehypertension was classified as having a systolic blood pressure between 120 and 139 mm Hg or diastolic blood pressure between 80 and 89 mm Hg and not receiving antihypertensive medications. HTN was defined as the occurrence of any of the following: systolic blood pressure ≥ 140 mm Hg or diastolic blood pressure ≥ 90 mm Hg or use of antihypertensive medications [24]. Dyslipidemia was defined as serum TG of ≥ 2.26 mmol/L or serum total cholesterol (TC) of ≥ 6.21 mmol/L or if the patient was taking lipid-lowering medications [25]. Smoking history was positive if patients reported a current or any previous history of smoking tobacco. American Diabetes Association criteria were used to define prediabetes and DM. Prediabetes was classified as HbA1c between 5.7 and 6.4% and not receiving antidiabetic medications while patients having HbA1c ≥ 6.5%, or on medications for diabetes were considered to have DM [26].

Patients were noted to have CHD if they had a documented history of a coronary event, coronary revascularization procedure, or a diagnosis established by a cardiologist. Similarly, patients were noted to have vascular disease if they had a documented history of cerebrovascular accident or transient ischemic attack, a documented history of peripheral arterial disease or revascularization for peripheral vascular disease.

### Outcomes

In this study, CKD stages 3–5 were defined using the National Kidney Foundation Kidney Disease Outcomes Quality Initiative guidelines as an eGFR < 60 mL/min/1.73 m^2^ for ≥ 3 months [27]. eGFR was calculated using the following CKD Epidemiology Collaboration (CKD-EPI) creatinine equation:

$$\begin{array}{l} \mathrm{eGFR}=141\times \min{\left(\mathrm{SCr}/\kappa ,1\right)}^{\alpha }\times \max{\left(\mathrm{SCr}/\kappa ,1\right)}^{-1.209}\times 0.993^{\mathrm{Age}\;}\times \left[1.018\;\mathrm{if}\;\mathrm{female}\right]\times \\ \left[1.159\;\mathrm{if}\;\mathrm{of}\;\mathrm{African}\;\mathrm{descent}\right],\;\mathrm{where}\;\mathrm{SCr}\;\mathrm{is}\;\mathrm{serum}\;\mathrm{creatinine}\;\mathrm{in}\;\mu \mathrm{mol}/L,\mathrm{age}\;\mathrm{is}\;\mathrm{in}\;\mathrm{years},\kappa =\\ 61.9\;\mathrm{for}\;\mathrm{females}\;\mathrm{and}\;79.6\;\mathrm{for}\;\mathrm{males},\mathrm{and}\;\alpha =\\ -0.329\;\mathrm{for}\;\mathrm{females}\;\mathrm{and}\;-0.411\\ \mathrm{for}\;\mathrm{males},\min\;\mathrm{is}\;\mathrm{the}\;\mathrm{minimum}\;\mathrm{of}\;\mathrm{SCr}/\kappa \;\mathrm{or}\;1,\mathrm{and}\;\max\;\mathrm{is}\;\mathrm{the}\;\mathrm{maximum}\;\mathrm{of}\;\mathrm{SCr}/\kappa \;\mathrm{or}\;1. \end{array}$$

In this equation, eGFR is expressed as mL/min/1.73m^2^. A factor of 1.0 was applied for ethnicity since no evidence was available for a correction factor related to the local population being studied, and there were no subjects of African descent [28].

All laboratory assays at baseline and follow-up were conducted at the Medical Laboratory Department of Tawam Hospital. Circulating levels of HbA1c were measured with an automated analyzer Integra 400 Plus (Roche Diagnostics, Mannheim, Germany). Lipid profile and serum creatinine were measured using standard methods with Synchron Clinical System (UniCel DxC-800; Beckman Coulter, Inc., Fullerton, CA, USA). Reference ranges for creatinine as suggested by the manufacturer were 53–115 mol/l and 58–96 μmol/l for males and females respectively.

### Statistical analyses

IBM SPSS version 25 was used to analyze the data. Distributions and categories were examined and categories with small sample size and skewed distributions were noted. Categories were meaningfully combined when indicated.

Baseline variables stratified by gender were tested for association between subjects who did and did not develop CKD stages 3–5 using independent-samples t-test for continuous variables and Fisher's exact test (two-tailed) for categorical variables.

Patient-years at risk for developing CKD stages 3–5 was calculated for each subject from the baseline visit in 2008 to the diagnosis of CKD stages 3–5 or last outpatient visit, whichever occurred first. The incidence rate was calculated as the number of new cases of CKD stage 3–5 divided by patient-years at risk.

Univariable analysis was performed on each predictor variable, age (categorized as ≤49, 50–64 and ≥65), gender (categorized as female and male), CHD (categorized as no and yes), DM (categorized as no and yes), vascular disease (categorized as no and yes), HTN (categorized as no and yes), dyslipidemia (categorized as no and yes), smoking (categorized as no and yes), and obesity (categorized as no and yes). The first category in the parenthesis in the above definition was defined as the reference group. Variables with a p-value <0.1 in the univariable analysis were included in the multivariable Cox's proportional hazards analysis. A backward stepwise method based on likelihood ratios was then used with entry and removal probabilities set at 0.05 and 0.10 respectively. Proportional hazards assumption was tested using log-log plot and was not significant. A p-value < 0.05 was considered significant.

## Results

### Baseline characteristics

Demographical, clinical, and biochemical data of the 491 subjects are presented in Table 1. The mean age at baseline was 53.20 ± 13.82 years, and half of the study subjects were males. Almost half of the subjects had a history of DM (215/491; 43.8%), and close to two-thirds had HTN (335/491; 68.2%). The mean baseline eGFR was 98.12 ± 18.50 mL/min/1.73 m^2^. Male subjects had higher history of CHD (13.2% vs 5.0%), vascular disease (8.8% vs 2.9%), and smoking (29.2% vs 0.8%), but had lower prevalence of obesity (40.4% vs 61.0%) than female subjects.

**Table 1:** Baseline characteristics of study subjects.

	Total (N = 491)	Males (N = 250)	Females (N = 241)
Age (years), Mean (SD)	53.20 ± 13.82	52.68 ± 15.30	53.75 ± 12.11
Male gender (%)	50.9	-	-
**History of (%)**			
CHD	9.2	13.2	5.0
DM	43.8	46.4	41.1
Vascular disease	5.9	8.8	2.9
HTN	68.2	68.8	67.6
Dyslipidemia	64.6	63.6	65.6
Smoking	15.3	29.2	0.8
Obesity	50.5	40.4	61.0
ACEI/ARB use	46.6	49.6	39.4
**Anthropometric values**			
BMI (kg/m^2^), Mean (SD)	30.19 ± 6.21	28.73 ± 5.77	31.7 ± 6.30
SBP (mmHg), Mean (SD)	131.37 ± 15.69	132.03 ± 15.65	130.70 ± 15.74
DBP (mmHg), Mean (SD)	76.87 ± 10.71	77.64 ± 11.34	76.07 ± 9.98
**Laboratory values**			
TC (mmol/L), Mean (SD)	4.98 ± 1.10	4.83 ± 1.09	5.13 ± 1.09
TG (mmol/L), Mean (SD)^a^	1.32 ± 0.79	1.35 ± 0.80	1.28 ± 0.78
Cr (μmol/l), Mean (SD)	67.86 ± 17.92	78.97 ± 16.00	56.33 ± 11.30
eGFR (mL/min/1.73m^2^), Mean (SD)	98.12 ± 18.50	95.56 ± 20.64	100.77 ± 15.60
HbA1c (%), Mean (SD)^b^	6.60 ± 1.71	6.77 ± 1.98	6.43 ± 1.39

CHD; Coronary heart disease, DM; diabetes mellitus, HTN; hypertension, ACEI; Angiotensin-converting enzyme inhibitors, ARB; Angiotensin II receptor blockers, BMI; Body mass index, eGFR; estimated glomerular filtration rate, SBP; Systolic blood pressure. DBP; Diastolic blood pressure, Cr; Creatinine, TC; Total cholesterol, TG; Triglycerides, SD; standard deviation, HbA1c; glycosylated Hemoglobin, Type A1C.

^a^ N = 485.

^b^ N = 476.

### Incidence

A total of 56 new cases (36 males and 20 females) of CKD stages 3–5 were identified after a mean follow-up of 8.6 years (95% CI 8.5, 8.8 years). The cumulative incidence of CKD stages 3–5 over this period was 11.4% (95% CI 8.6, 14.2). The incidence rate of CKD stages 3–5 was 164.8 (95% CI 121.6, 207.9) cases per 10,000 person-years.

### Analyses of risk factors

The characteristics of subjects stratified by gender that developed CKD stages 3–5 compared with those who did not develop the disease are shown in S1 Table. In males, subjects who developed CKD stages 3–5 were older and had a history of CHD, DM, vascular disease, HTN, and dyslipidemia compared with those who did not develop CKD stages 3–5. Among female subjects, older age, DM, HTN, and obesity were risk factors that were associated with CKD stages 3–5.

**Fig 2:**
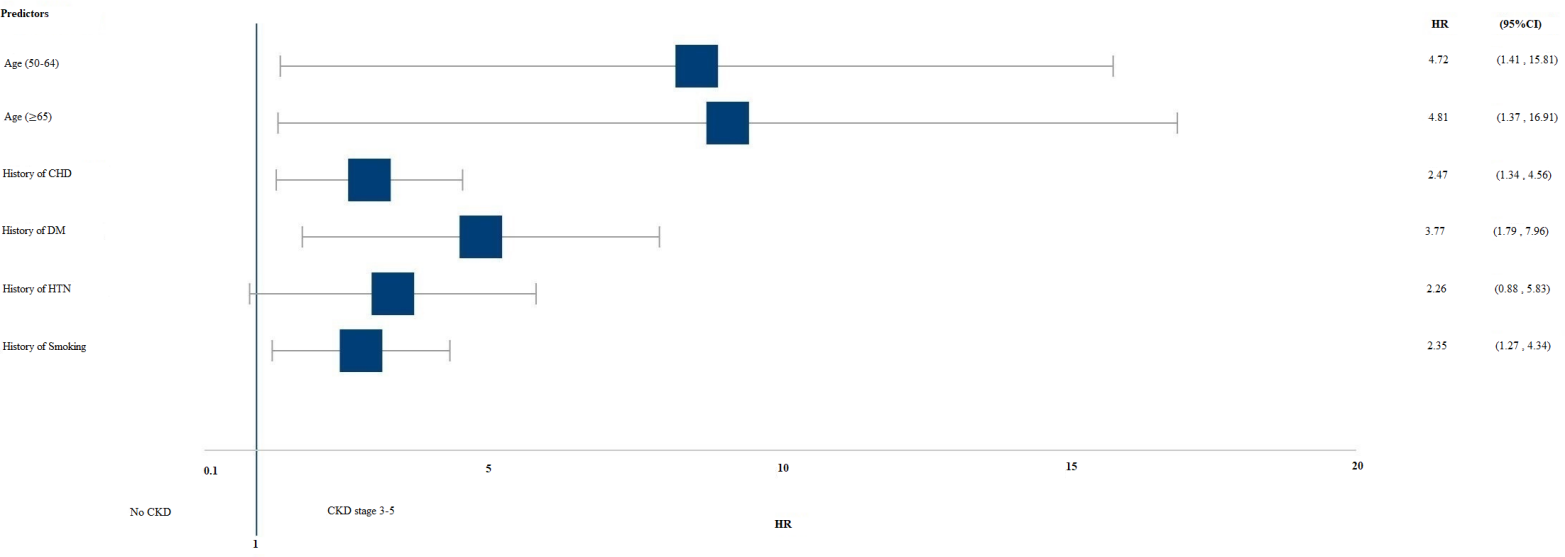
Adjusted Hazards ratio (HR) of risk factors associated with developing CKD stages 3–5. CKD; Chronic kidney disease, CHD; Coronary heart disease, HTN; hypertension, DM; diabetes mellitus. HR was adjusted for age, gender, history of CHD, history of DM, history of vascular disease, history of HTN, history of dyslipidemia, and history of smoking in a Cox HR proportional model.

Table 2 and Fig 2 show the adjusted hazard ratios (HR) of risk factors associated with developing CKD stages 3–5. Subjects with a history of CHD were 2.5 times more likely to develop CKD stages 3–5 than those with no history of CHD, and patients with diabetes had almost quadruple the risk compared with those without diabetes. History of smoking increased the risk of CKD stages 3–5 by 2.4 times compared with no history of smoking.

**Table 2:** Unadjusted and adjusted^a^ Hazards ratios (HR) and 95% confidence intervals (95% CI) of predictors for developing CKD stages 3–5.

Predictor Variables	Univariable (N = 485)^b^		Multivariable (N = 485)^b^	
	Unadjusted	p-value	Adjusted^a^	p-value
	HR (95% CI)		HR (95% CI)	
**Age**				
≤49	1.00		1.00	
50–64	8.82(2.69–28.89)	<0.001	4.72(1.41–15.81)	0.012
≥65	12.37(3.70–41.33)	<0.001	4.81(1.37–16.91)	0.014
**Gender**				
Females	1.00		1.00	
Males	1.90(1.09–3.28)	0.023	1.25(0.66–2.36)	0.488
**History of CHD**				
No	1.00		1.00	
Yes	5.41(3.05–9.59)	<0.001	2.47(1.34–4.56)	0.004
**History of DM**				
No	1.00		1.00	
Yes	7.07(3.46–14.45)	<0.001	3.77(1.79–7.96)	0.001
**History of Vascular disease**				
No	1.00		1.00	
Yes	2.52(1.14–5.58)	0.022	1.70(0.40–7.23)	0.472
**History of HTN**				
No	1.00		1.00	
Yes	4.63(1.84–11.60)	0.001	2.26(0.88–5.83)	0.092
**History of Dyslipidemia**				
No	1.00		1.00	
Yes	2.82(1.38–5.76)	0.004	0.99(0.46–2.13)	0.988
**History of Smoking**				
No	1.00		1.00	
Yes	2.18(1.19–4.00)	0.012	2.35(1.27–4.34)	0.006
**History of Obesity**				
No	1.00		Not applicable^c^	
Yes	1.15(0.68–1.96)	0.602		

CKD; Chronic kidney disease, CHD; Coronary heart disease, DM; diabetes mellitus, HTN; hypertension.

^a^ Multivariable Cox model, adjusted for all predictors in the final model selected using backward selection.

^b^ Six cases were excluded due to being censored before the earliest event in a stratum.

^c^ P-value > 0.1 in the initial univariable analysis and was not included in the multivariable analysis.

## Discussion

### Incidence

The results from our study showed that the incidence of CKD stages 3–5, in high-risk UAE nationals was more than 1% per year, during a 9-year period. The incidence rate of CKD stages 3–5 was 164.8 per 10,000 person-years. Surprisingly, the incidence rate of these CKD stages in our study was lower than expected. A Spanish prospective cohort study of 3,443 people with type 2 DM found that the incidence rate of CKD stages 3–5 is 207 per 10,000 person-years [29]. In a US study of native Pima Indians with type 2 DM, the incidence rates of CKD stages 3–5 are 254 and 219 per 10,000 person-years in men and women, respectively [30]. Higher incidence rates in these studies could be explained by the increased prevalence of diabetes in their sample population. Conversely, a large community-based cohort study from Iran, in contrast to our study subjects, had a lower prevalence of diabetes mellitus and showed a higher incidence rate of CKD stages 3–5 (214.82 per 10,000 person-years) [31]. Another plausible explanation for the lower incidence rate of CKD stage 3–5 could be due to CVD related deaths which were unaccounted for in the present study.

### Risk factors

In our study population, age, history of CHD, history of DM, and history of smoking were significant risk factors for developing CKD stages 3–5. In several studies, CHD has been found to be an independent risk factor for developing CKD [29,32,33]. However, in the few studies conducted among Middle Eastern and Asian populations, no association was shown between CHD and CKD [31,34]. In our study, history of CHD showed an adjusted HR of 2.47 (95% CI, 1.34, 4.56; p = 0.004).

Diabetes is an important and established risk factor for the development of CKD [5,6,29,31,34–36]. Our findings also showed that diabetes was a very strong predictor for the development of CKD stages 3–5. In a recent study of 6,251 US adults with diabetes, the presence and duration of the disease are directly related to the prevalence of CKD [37]. Therefore, it is particularly important that local health care providers make diabetes prevention a priority.

Smoking is a known independent risk factor for the development of CKD [6,38,39], and our study also showed that smoking was a statistically significant risk factor. Some studies went further to document an association with the number of pack-years smoked and CKD [39,40]. Prior research on the effects of smoking and CKD has been instrumental in recommending and developing smoking cessation programs as part of a comprehensive CKD prevention and management strategy [38–40].

HTN is a recognized risk factor for CKD. Numerous studies have identified a strong association between high blood pressure and CKD [6,29,41]. However, we found that the association between HTN and development of CKD stages 3–5 was marginally significant. Approximately 50% of males and 40% of females in our study were on angiotensin-converting enzyme (ACE) inhibitors or angiotensin II receptor blockers (ARBs), which delay the progression of CKD [42]. Therefore, the nephroprotective effects of these medications on patients susceptible to CKD could account for the marginally significant result. However, this does not preclude HTN as a clinically significant and well-recognized modifiable risk factor for CKD.

Blood pressure control is the single most important intervention to reduce the development of CKD in high-risk patients [43], while the control of glycemia has been shown to be a major factor in the prevention of diabetic nephropathy [44,45]. Further to this, the use of ACE inhibitors and ARBs has been widely advocated as pharmacologic prevention and treatment of CKD in patients with diabetes, particularly in the presence of albuminuria [46]. Despite this therapeutic advantage, a study showed that 25% of physicians in the UAE did not use ACE inhibitors to treat patients with CKD and nearly half of patients prescribed ACE inhibitors were non-compliant [4]. Strategies that optimize therapy and enhance compliance need to be adopted by local healthcare authorities to prevent the development and progression of CKD in high-risk patients.

### Limitations and strengths

This study has several limitations. First, the data quality and standardization of laboratory and anthropometric measurements in a retrospective study may not be as good as in a prospective study. Second, patient mortality in this study was unaccounted for, therefore, the incidence of CKD may be underestimated. Third, eGFR was calculated using the CKD-EPI equation, which has been validated in Caucasian and African American populations; there are limited available data for people of Arab descent [47]. Finally, the changes in variables such as blood pressure and HbA1c, and the impact of nephroprotective medications such as ACE inhibitors and ARBs over time were not evaluated in this study. Further studies are needed to explore the effect of changes in these variables and their relationship on CKD in this population.

As for strengths, our case definition of CKD does not account for albuminuria. It is not uncommon for people to regress from moderate to normoalbuminuria naturally or as a result of treatment [30]. Strictly limiting the definition of CKD to eGFR readings ensures that our results are not influenced by reversible kidney injury. Furthermore, the diagnosis of CKD stages 3–5 was based on two consecutive readings of eGFR < 60 mL/min/1.73 m^2^, taken more than or equal to three months apart. By doing so, this study accounts for the intra-individual variability in eGFR. Both these measures in our study lead to a more accurate representation of kidney function. Moreover, this study used documented anthropometric measurements and laboratory data for risk factor classification rather than self-reported information. Finally, we used the CKD-EPI equation to define CKD stages 3–5; CKD-EPI equation estimates measured GFR more accurately than the modification of diet in renal disease study equation in most studies [28,48,49].

## Conclusions

The cumulative incidence of CKD stages 3–5 among high-risk UAE nationals was 11.4% over a 9-year period. The major risk factors identified were older age, history of CHD, DM, and history of smoking. Evidence-based strategies for prevention of CHD and diabetes and smoking cessation programs could lower the risk of developing CKD stages 3–5 among the UAE national population with or at high-risk of CVD.

## Supporting information

S1 Table Comparison of baseline characteristics stratified by gender and of the whole population according to development of CKD stages 3–5. CKD; Chronic kidney disease, CHD; Coronary heart disease, DM; diabetes mellitus, HTN; hypertension, ACEI; Angiotensin-converting enzyme inhibitors, ARB; Angiotensin II receptor blockers, BMI; Body mass index, eGFR; estimated glomerular filtration rate, SBP; Systolic blood pressure. DBP; Diastolic blood pressure, Cr; Creatinine, TC; Total cholesterol, TG; Triglycerides, SD; standard deviation, HbA1c; glycosylated Hemoglobin, Type A1C.
(DOCX)

S1 Dataset Chronic kidney disease in patients at high risk of cardiovascular disease in the United Arab Emirates: A population-based study dataset. ID; identification, CHD; Coronary heart disease, HTN; hypertension, DLD; dyslipidemia, DM; diabetes mellitus, Angiotensin-converting enzyme inhibitors, ARB; Angiotensin II receptor blockers, HbA1c; glycosylated Hemoglobin, estimated glomerular filtration rate, SBP; Systolic blood pressure. DBP; Diastolic blood pressure, BMI; Body mass index, CKD; Chronic kidney disease.
(XLSX)
