# Supplementary material for: Chronic kidney disease in patients at high risk of cardiovascular disease in the United Arab Emirates: A population-based study
Source: PLoS One. 2018 Jun 27;13(6):e0199920. doi: 10.1371/journal.pone.0199920 (PMC6021088; doi:10.1371/journal.pone.0199920)
Supplement: S1 Table — CKD; Chronic kidney disease, CHD; Coronary heart disease, DM; diabetes mellitus, HTN; hypertension, ACEI; Angiotensin-converting enzyme inhibitors, ARB; Angiotensin II receptor blockers, BMI; Body mass index, eGFR; estimated glomerular filtration rate, SBP; Systolic blood pressure. DBP; Diastolic blood pressure, Cr; Creatinine, TC; Total cholesterol, TG; Triglycerides, SD; standard deviation, HbA1c; glycosylated Hemoglobin, Type A1C. (DOCX) [file pone.0199920.s001.docx]

**S1 Table. Comparison of baseline characteristics stratified by gender and of the whole population according to development of CKD stages 3-5.**

|  | **Males** | | | | **Females** | | | | **Total** | | | |
| --- | --- | --- | --- | --- | --- | --- | --- | --- | --- | --- | --- | --- |
|  |  | **CKD^a^**  **(N=36)** | **No CKD (N=214)** | **p-value^b^** |  | **CKD^a^**  **(N=20)** | **No CKD (N=221)** | **p-value^b^** |  | **CKD^a^**  **(N=56)** | **No CKD**  **(N=435)** | **p-value^b^** |
| Age (years), Mean (SD) |  | 62.53(10.37) | 51.02(15.39) | <0.001 |  | 61.70(7.97) | 53.03(12.18) | 0.002 |  | 62.23(9.51) | 52.04(13.87) | <0.001 |
| Male gender (%) |  | - | - | - |  | - | - | - |  | 64.3 | 49.2 | 0.046 |
| **History of** (%) |  |  |  |  |  |  |  |  |  |  |  |  |
| CHD |  | 38.9 | 8.9 | <0.001 |  | 15.0 | 4.1 | 0.066 |  | 30.4 | 6.4 | <0.001 |
| DM |  | 80.6 | 40.7 | <0.001 |  | 90.0 | 36.7 | <0.001 |  | 83.9 | 38.6 | <0.001 |
| Vascular disease |  | 19.4 | 7.0 | 0.024 |  | 0.0 | 3.2 | 1.000 |  | 12.5 | 5.1 | 0.036 |
| HTN |  | 88.9 | 65.4 | 0.006 |  | 95.0 | 65.2 | 0.005 |  | 91.1 | 65.3 | <0.001 |
| Dyslipidemia |  | 83.3 | 60.3 | 0.008 |  | 85.0 | 63.8 | 0.083 |  | 83.9 | 62.1 | 0.001 |
| Smoking |  | 38.9 | 27.6 | 0.171 |  | 0.0 | 0.9 | 1.000 |  | 25.0 | 14.0 | 0.046 |
| Obesity |  | 38.9 | 40.7 | 1.000 |  | 85.0 | 58.8 | 0.029 |  | 55.4 | 49.9 | 0.480 |
| ACEI/ARB use |  | 75.0 | 45.3 | 0.001 |  | 80.0 | 35.7 | <0.001 |  | 76.8 | 40.5 | <0.001 |
| **Anthropometric values** |  |  |  |  |  |  |  |  |  |  |  |  |
| BMI (kg/m^2^), Mean (SD) |  | 28.10(5.76) | 28.83(5.78) | 0.481 |  | 33.43(4.98) | 31.54(6.39) | 0.200 |  | 30.00(6.03) | 30.21(6.24) | 0.814 |
| SBP (mmHg), Mean (SD) |  | 136.47(16.81) | 131.28(15.36) | 0.065 |  | 137.20(19.54) | 130.11(15.27) | 0.053 |  | 136.73(17.66) | 130.69(15.31) | 0.007 |
| DBP (mmHg), Mean (SD) |  | 74.61(12.65) | 78.15(11.05) | 0.083 |  | 75.00(10.50) | 76.17(9.95) | 0.617 |  | 74.75(11.83) | 77.14(10.54) | 0.115 |
| **Laboratory values** |  |  |  |  |  |  |  |  |  |  |  |  |
| TC (mmol/L), Mean (SD) |  | 4.48(1.15) | 4.89(1.07) | 0.034 |  | 4.66(1.0) | 5.17(1.09) | 0.044 |  | 4.54(1.09) | 5.03(1.09) | 0.002 |
| TG (mmol/L), Mean (SD)^c^ |  | 1.51(0.71) | 1.32(0.81) | 0.188 |  | 1.49(0.75) | 1.26(0.79) | 0.213 |  | 1.50(0.72) | 1.29(0.80) | 0.058 |
| Cr (µmol/l), Mean (SD) |  | 91.89(12.03) | 76.80(15.58) | <0.001 |  | 66.50(11.89) | 55.40(10.80) | <0.001 |  | 82.82(17.08) | 65.93(17.12) | <0.001 |
| eGFR (mL/min/1.73m^2^), Mean (SD) |  | 77.15(11.88) | 98.66(20.19) | <0.001 |  | 84.08(12.57) | 102.28(14.97) | <0.001 |  | 79.62(12.48) | 100.50(17.80) | <0.001 |
| HbA1c (%), Mean (SD)^d^ |  | 8.52(2.89) | 6.47(1.62) | <0.001 |  | 7.92(1.92) | 6.29(1.25) | 0.001 |  | 8.30(2.57) | 6.38(1.44) | <0.001 |

^a^CKD stages 3-5.

^b^Independent-samples t-test was used to calculate p values for continuous variables and Fisher's exact test (two-tailed) for categorical variables.

^c^N=485.

^d^N=476.
